# An easier and safe affair, pleural drainage with ultrasound in critical patient: a technical note

**DOI:** 10.1186/s13089-018-0098-z

**Published:** 2018-08-01

**Authors:** Luigi Vetrugno, Giovanni Maria Guadagnin, Daniele Orso, Enrico Boero, Elena Bignami, Tiziana Bove

**Affiliations:** 1 Anesthesiology and Intensive Care Clinic, Department of Medicine, University of Udine, P.le S. Maria della Misericordia n.15, 33100 Udine, Italy; 2 Anesthesiology and Intensive Care, Department of Surgical Sciences, University of Turin, Turin, Italy; 3 Anesthesiology, Critical Care and Pain Medicine Division, Department of Medicine and Surgery, University of Parma, Parma, Italy

**Keywords:** Pleural effusion, Thoracic ultrasound, Ultrasound guidance, Pleural drainage

## Abstract

Thoracic ultrasound is a powerful diagnostic imaging technique for pleural space disorders. In addition to visualising pleural effusion, thoracic ultrasound also helps clinicians to identify the best puncture site and to guide the drainage insertion procedure. Thoracic ultrasound is essential during these invasive manoeuvres to increase safety and decrease potential life-threatening complications. This paper provides a technical description of pigtail-type drainage insertion using thoracic ultrasound, paying particular attention to indications, contraindications, ultrasound guidance, preparation/equipment, procedure and complications.

**Electronic supplementary material:**

The online version of this article (10.1186/s13089-018-0098-z) contains supplementary material, which is available to authorized users.

## Background

Percutaneous pleural drainage is the third most commonly performed procedure in the intensive care unit (ICU) after vascular catheterisation and tracheal intubation [1–3]. Forty-one percent of patients admitted to the ICU have pleural effusion at the time of admission, while 21% will develop it during hospitalisation [4]. The gold standard technique for pleural effusion (PLEFF) diagnosis is computed tomography (CT), which requires transporting critical patients outside the ICU [5]. Thoracic ultrasound (TUS) allows a bedside approach for PLEFF diagnosis with a sensitivity of 92%, a specificity of 93% and a diagnostic accuracy of 93% [6], thus showing better reliability and accuracy than chest X-ray, without using ionising radiation. The use of bedside ultrasound (US) not only leads to an improvement in the diagnosis [7], but also allows the detection of the best puncture site and the fluid quantification of PLEFF [8, 9]. The positioning of percutaneous pleural drainage with TUS guidance increases the procedure’s success rate and safety [10]. International guidelines recommend ultrasound guidance for pleural drainage procedures and the usage of small-bore catheters [11]. Our technical note describes the pigtail insertion using ultrasound, paying particular attention to indications, contraindications, preparation/equipment, ultrasound guidance, procedure and complications.

## Indications

Pleural effusions drainage theoretically improves oxygenation by enhancing the ventilation–perfusion ratio and by reducing arteriovenous shunt, re-expanding areas of a collapsed, poorly ventilated lung [4]. Consequently, drainage of PLEFF seems to accelerate the weaning process from mechanical ventilation [4]. Respiratory mechanics could significantly improve after effusion drainage because it leads to an increase in end-expiratory transpulmonary pressure, respiratory system compliance, end-expiratory lung volume and a decrease in plateau pressure [12]. Pleural drainage also provides a chance to obtain chemical-physical-cytological samples to guide the differential diagnosis of PLEFF and any follow-up therapy. Indications [11, 13] and relative contraindications [14] are shown in Table 1.

**Table 1:** Indications and contraindications to pleural drainage positioning

Indications	Contraindications (relative)
Recurrent malignant pleural effusion Symptomatic patients caused by the effusions Massive transudative or exsudative pleural effusion Parapneumonic effusions Weaning from mechanical ventilation	Coagulopathy, thrombocytopenia Small to medium size pleural effusions in cardiac patients with more than moderate left ventricular dysfunction Pulmonary bullae Pulmonary, pleural or thoracic adhesions Loculated pleural effusion or empyema Recurrent pleural infections Skin infection over the chest tube insertion site

## Preparation and equipment

The position of the patients is dependent on the operator preference [11]. To increase safety margin (depth of pleural effusion) [15, 16], whenever possible, place the patient in supine position with trunk elevation of 40–45° (in this position effusion gravitates down to the lower part of the chest pushing up the lung) and with arm elevated behind the head. However, as critically ill patients may often have limited mobility and also upright sitting position can generate haemodynamic side-effects (i.e., requirements of vasopressors) in a short time, the procedure will be described in a supine rather than a seated position. Considering that, some patients are limited ability to elevate the arm above the shoulder; it can be fixed in a soft bandage to the other side of the bed in a direction to the opposite shoulder.

**Fig. 1:**
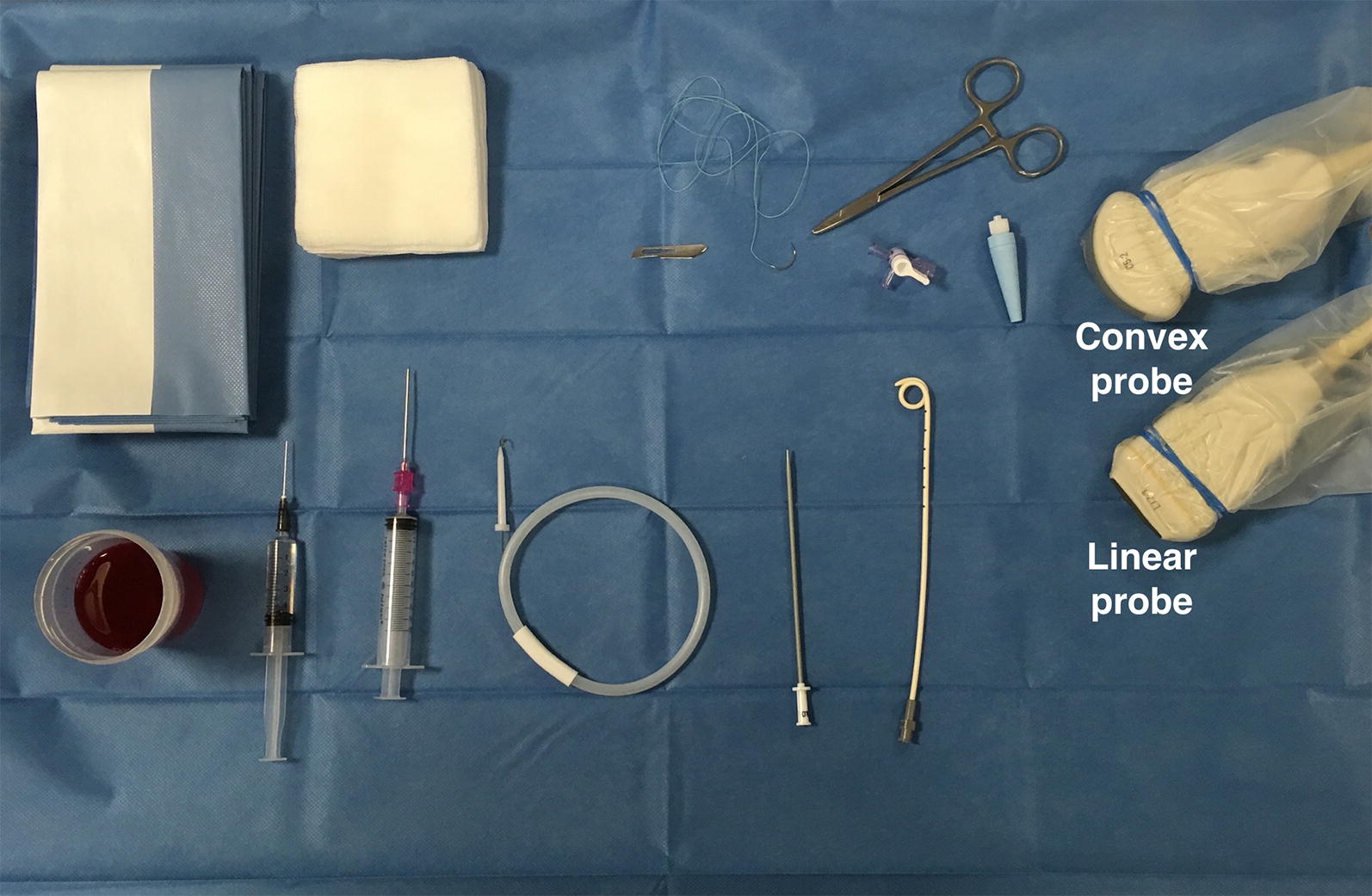
A set of pleural drainage tools on the tray

Never rotate patient to the opposite site to create room for the procedure, as the fluid moves towards the paravertebral zone. The puncture might be more dangerous and patient could then lie on the insertion site which relates to infection complications. In patients with mechanical ventilation, do not take down PEEP or disconnect patient during drainage insertion because those manoeuvers can be associated with severe alveolar de-recruitment, especially in a patient that required high mean airway pressure and high PEEP to expand a poor compliance lung. On the other hand, the possibility of sudden reduction of intrapleural pressure due to puncture with risk of puncture of the visceral pleura is unlikely, especially in the case of ultrasound-guided puncture. Pigtail insertion is a sterile procedure; consequently, the operator should wear sterile gloves and use sterile drapes, sterilised materials on a sterilised puncture site. The necessary materials are listed in Table 2 and shown in Fig. 1. About pain management, pleura could be the most painful portion of the procedure, as it is highly innervated. For patients who are intubated you have the benefits of general anesthesia. For awake patient, pleural drainage insertion can be performed in a pain free manner if local anesthesia is performed properly, especially just above the upper rib edge.

**Table 2:** Percutaneous pigtail drainage insertion equipment

For the operator	Over the tray	Patient
Medical hat	Sterile drapes	Disinfection of puncture site
Medical mask	Sterile towels	Sterile drapes
Hand disinfection	Syringes (5–10 mL)	Informed consent (when possible)
Sterile gown	Local anaesthetics	
Sterile gloves	Sterile water	
	Phased array probe or convex and linear probe Sterile US probe cover	
	Introducer needle	
	Guidewire	
	Scalpel and dilator	
	Catheters (8–14F)	
	Trocar	
	Suture and medical dressing	

*US* ultrasound, *F* French (1F = 0.33 mm)

## Ultrasound guidance

### Identification of puncture site

Pigtail insertion should be carried out in the *safety triangle,* almost always at the posterior axillary line if aiming for effusion [11], and performed under image guidance [11, 17]. The safety triangle is bordered by the lateral edge of the *pectoralis major*, the lateral edge of the *latissimus dorsi* and a line along the fifth intercostal space at the level of the nipple. However, ultrasound guidance allows the operator to better decide where to insert the pigtail. The best puncture site is, in fact, the place where the operator can visualise each anatomical structure (i.e., diaphragm, pleural, and organs) and where the operator can measure the maximum distance between visceral and parietal pleural (increasing the safety margin). One of the most commonly used US method for estimation of pleural effusion volume (*V*) is represented by the Balik equation: *V* (mL) = 20 × Sep (mm) [8]. To obtain separation (Sep), the operator has to measure the maximal vertical distance between the parietal and visceral pleura in end-expiration at the lung base, in supine patient with trunk elevation of 10–15°. The patient’s position greatly influences the extent of PLEFF [18]. In addition, for pleural effusion when measuring the maximal end-expiratory distance between the parietal and visceral pleura at the thoracic base the area under the curve, as reported by Vignon et al. [19], was greater for right-sided pleural effusions, on the left side the heart increases the fluid level, like a stone in a water recipient. The operator should also examine the ultrasound features of the effusion; the presence of hyperechoic material within the effusion indicates an exsudative effusion.

### Intercostal artery visualisation

**Fig. 2:**
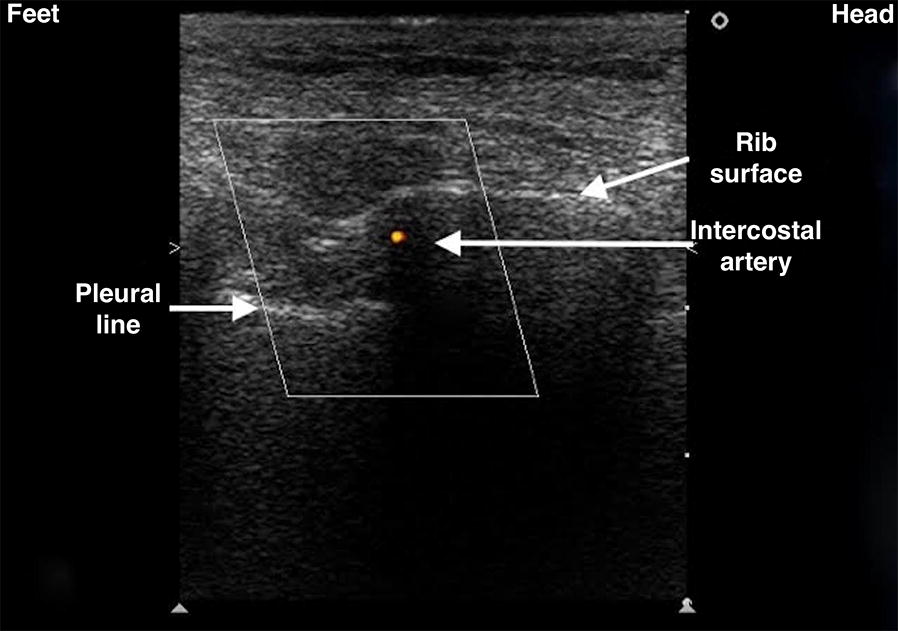
Visualisation of intercostal vessels using Doppler ultrasound. The probe marker is facing the patient’s head (on the right of the screen). The colour Doppler box should be placed on the bottom edge of the rib and the depth reduced until the rib does not occupy the entire screen. Pulse repetition frequency (PRF) should be reduced until pulsation is detected

It was once thought that ultrasound was incapable of identifying intercostal vessels [10]. However, some studies have shown that Doppler ultrasound can be used to visualise intercostal vessels [20–23] helping prevent vessel injury and ensure a procedure with a low risk of bleeding, even in patients with abnormal pre-procedural coagulation parameters [24]. The colour Doppler box should be placed on the bottom edge of the rib and the depth reduced so the rib does not occupy the entire screen. Reduce the pulse repetition frequency (PRF) until pulsation is detected (Fig. 2, Additional file 1: Video S1). Take care the more posteriorly puncture site is performed, the greater the risk of puncture of the intercostal artery which is not covered by the lower rib edge approaching to the paravertebral line [25–28].

### Site marking or direct needle guidance

Ultrasound guidance during pigtail insertion is performed using either *site marking* or *direct needle guidance* [29]. In the first case, the physician defines the optimal location point and marks it on the skin, then carries out the procedure without using real-time visualisation through US. Remember that patient repositioning can lead to fluid reallocation, so drain insertion has to be performed instantly after “*site marking”*. In “*direct needle guidance*” ultrasound is used in real-time to guide the pleural puncture; some operators prefer to perform needle insertion under real-time ultrasound guidance even though this approach is more technically challenging [29]. Advantages and limitations of ultrasound guidance are enlisted in Table 3.

**Table 3:** Advantages and limitations of ultrasound guidance

Advantage	Limitations
Can be performed in any position	Thickness of ribcage and soft tissues (i.e., obese patients)
Identification of the best site of puncture and best safety margin	Subcutaneous emphysema or large thoracic dressings

## The procedure

The following steps may also be performed with a single phased array probe, according to the local transducers availability. In the case of a single transducer the point IV will be performed without changing the probe.

**Fig. 3:**
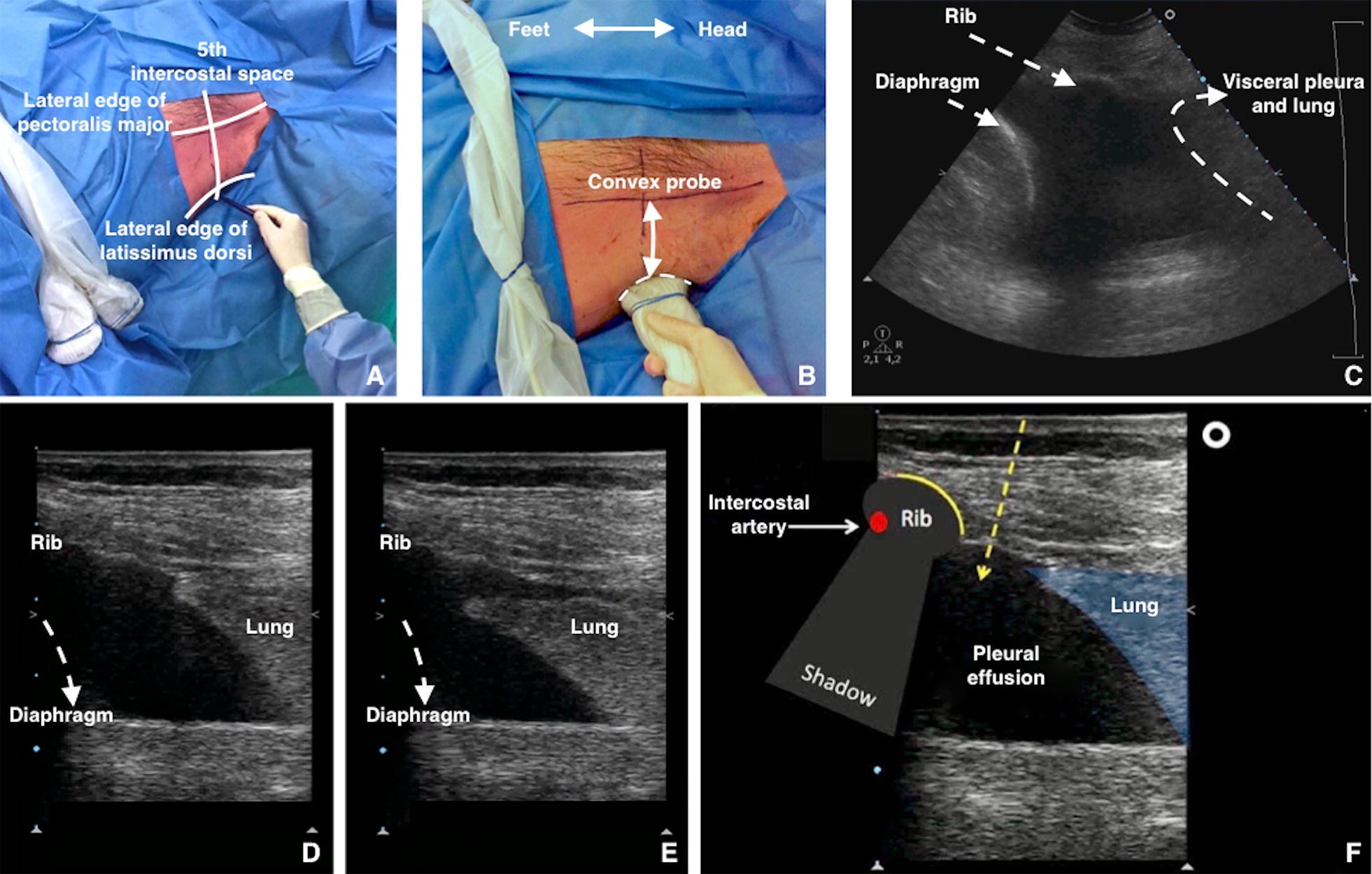
**A** Identify and draw the safety triangle. Note both convex and linear probes are present. **B**, **C** Use a low-frequency US transducer (convex or phased array probe) to identify the best puncture site. The best puncture site is where the operator can visualise each anatomical structures (i.e., diaphragm, pleural, organs) and can measure the maximum distance between visceral and parietal pleural (increasing the safety margin). The probe should be used in the transverse position between two ribs. The probe marker is facing the patient’s head (on the right of the screen). **D** At the end-expiration (high-frequency US transducer) diaphragm reaches the most cranial position. **E** At the end inspiration (high-frequency US transducer) lung reaches the most caudal position. **F** A high-frequency US transducer (linear probe) should be used in the transverse position, between two ribs to understand the upper and lower border of the needle insertion area. The probe marker is facing the patient’s head (on the right of the screen). The operator designs the course of the needle within the expected insertion area

**Fig. 4:**
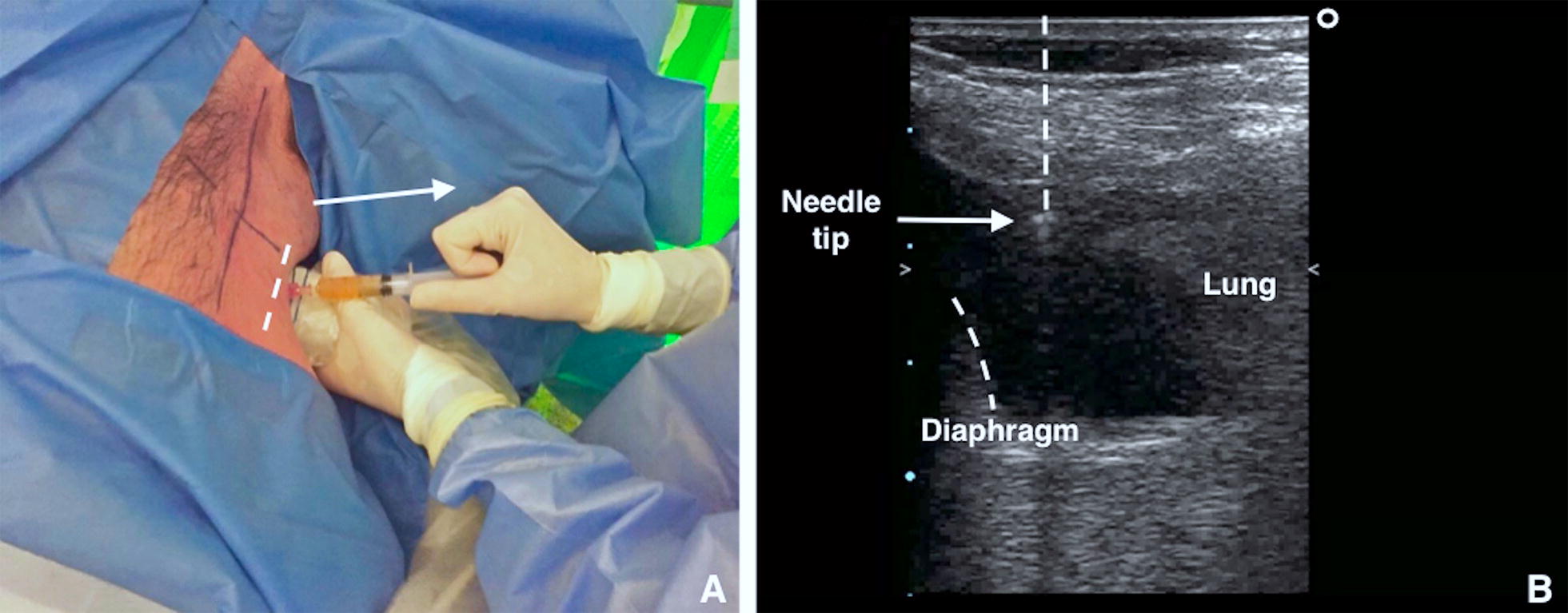
**A** Using the high-frequency US transducer (linear probe) in the transverse position, the puncture is performed employing short axis ultrasound-guidance (out of plane technique) at the point previously identified. Aspiration of fluid with a syringe confirms correct position of the needle tip. **B** With direct needle guidance, the correct position of the needle tip is visualised in real-time and is monitored constantly
C. Guidewire insertion and guidewire position check. Remove the syringe from the needle and pass the guidewire through the needle. Then remove the needle, leaving the guidewire in place (Fig. 5). It is mandatory to define the final position of the guidewire using US prior to proceeding with dilation (Fig. 5, Additional file 3: Video S3). It can be visualised as a hyperechoic linear structure from the insertion point at the skin surface to the hypoechoic effusion. Vertical rotation of the probe over the intercostal space allows for visualisation of the guidewire (leading towards the costophrenic space). Check that the guidewire is moving freely in and out of the dilator throughout this process to avoid kinking the guidewire.

A. Identify the best site for the puncture. Use a low-frequency (3.5–5 MHz) ultrasound transducer *(convex or phased array probe)* to identify the best puncture site evaluating these following steps. The probe should be used in the transverse position between two ribs (Fig. 3, Additional file 2: Video S2):

  I. *Diaphragm.* The position of the diaphragm should always be marked at end-expiration when it is the most cranial in position;
  II. *Organs.* Subdiaphragmatic (liver, spleen, kidneys) and over-diaphragmatic (visceral and parietal pleura, heart, lungs);
  III. *Maximum distance between visceral and parietal pleura*. This increases the safety margin;
  IV. *Shift to the high*-*frequency* (7–15 MHz) *ultrasound transducer* (linear probe). The probe should be used in the transverse position between two ribs to identify the upper and lower borders of the needle insertion area. The puncture site and the needle trajectory must be carefully designed (Fig. 3), with particular attention to the depth required to reach the pleural fluid and avoid lung injuries. In morbidly obese with limited rib palpation, linear probe can also be used to help to be perpendicular and over the upper rib edge with great safety.
  V. *Intercostal artery*.
B. Ultrasound-guided puncture. To minimize the risk of neurovascular bundle injury the needle must aim to the upper rib margin perpendicular to pleura, with the transducer to follow the needle trajectory under direct needle guidance (Fig. 4). The needle must be advanced slowly under direct visualisation. Aspiration of fluid with a syringe confirms the correct position using the site marking technique. For direct needle guidance, the correct position of the needle tip is visualised in real-time and constantly monitored (Fig. 4).

**Fig. 5:**
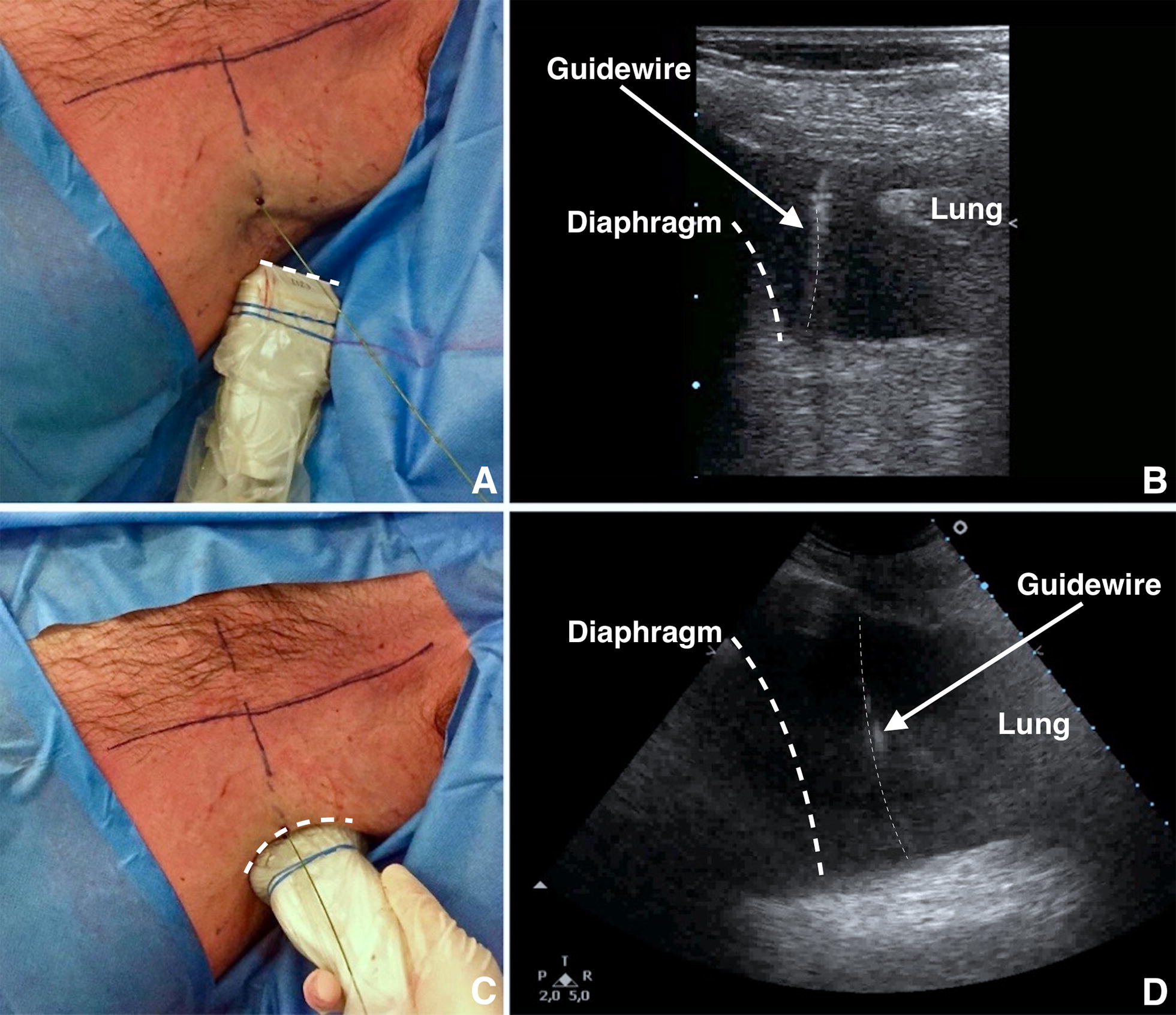
**A** Remove the syringe from the needle and pass the guidewire through the needle. After inserting the guidewire, remove the needle, leaving the guidewire in place. **B** Employing a high-frequency US transducer (linear probe) it is possible to visualise the insertion of the guidewire in real-time. **C** Shift to low-frequency US transducer (convex probe) to define the final position of the guidewire using US. It is mandatory to check the correct insertion of the guidewire at the end of the procedure. **D** As shown, the guidewire is correctly positioned within pleural effusion
D. Dilation. Make a small incision adjacent to the guidewire with the scalpel, then pass the dilator through the guidewire into the pleural space (Fig. 6). The dilator should not be introduced further than 1 cm beyond the depth from skin to parietal pleura; excessive dilator insertion increases the risk of visceral injury [24]; this depth can be safely taken with ultrasound.

**Fig. 6:**
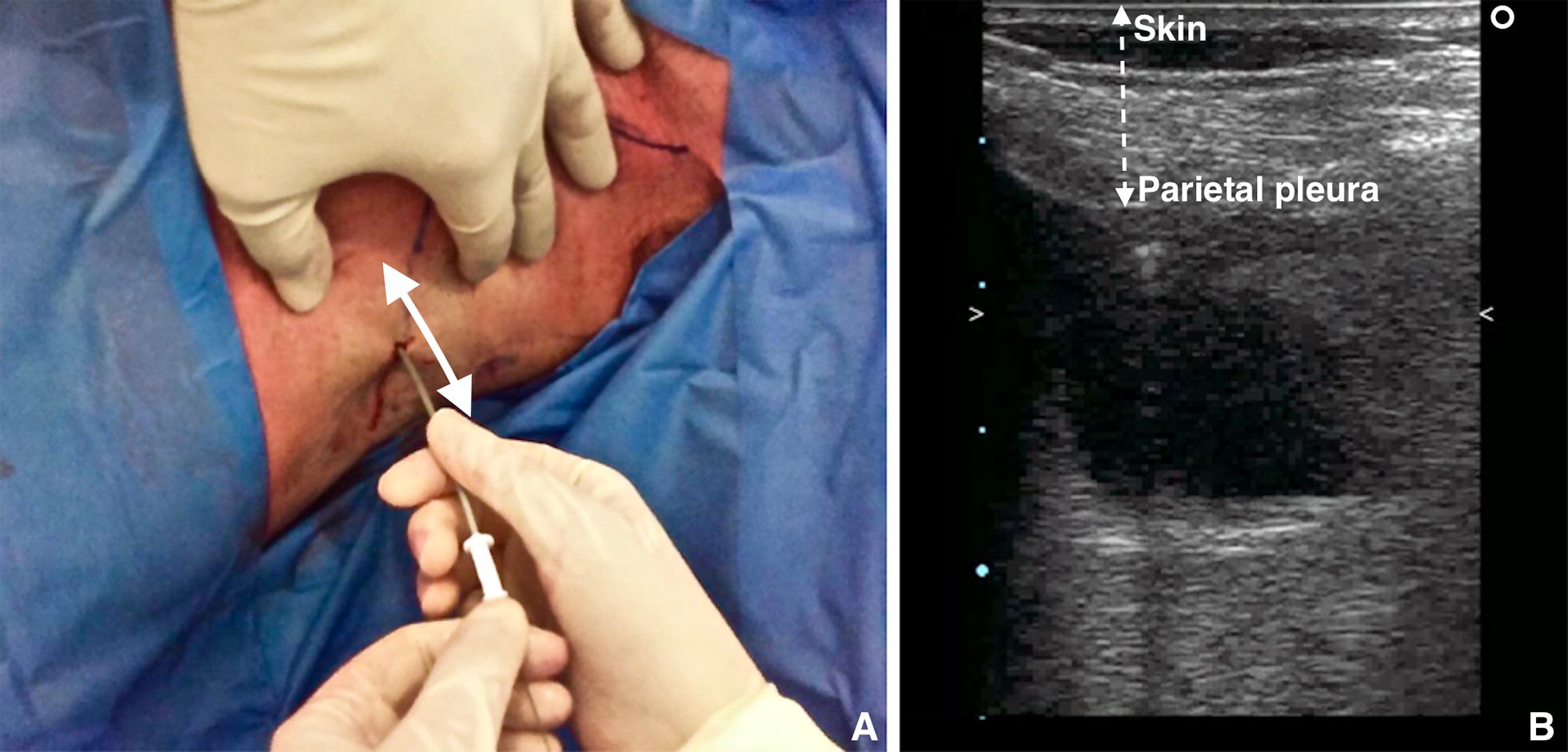
**A** Using the high-frequency probe, it is possible to calculate the distance between the skin and parietal pleura. **B** The dilator should not be inserted further than 1 cm beyond the depth from skin to pleural space
E. Pigtail insertion. The pigtail is passed over the guidewire, making sure that the last side hole is within the pleural space. Remove the guidewire, leaving the pigtail catheter in place (Fig. 7). After the guidewire has been removed, the drain is connected to the drainage system. Suture the pigtail to the chest wall in a manner similar to conventional chest tubes.

**Fig. 7:**
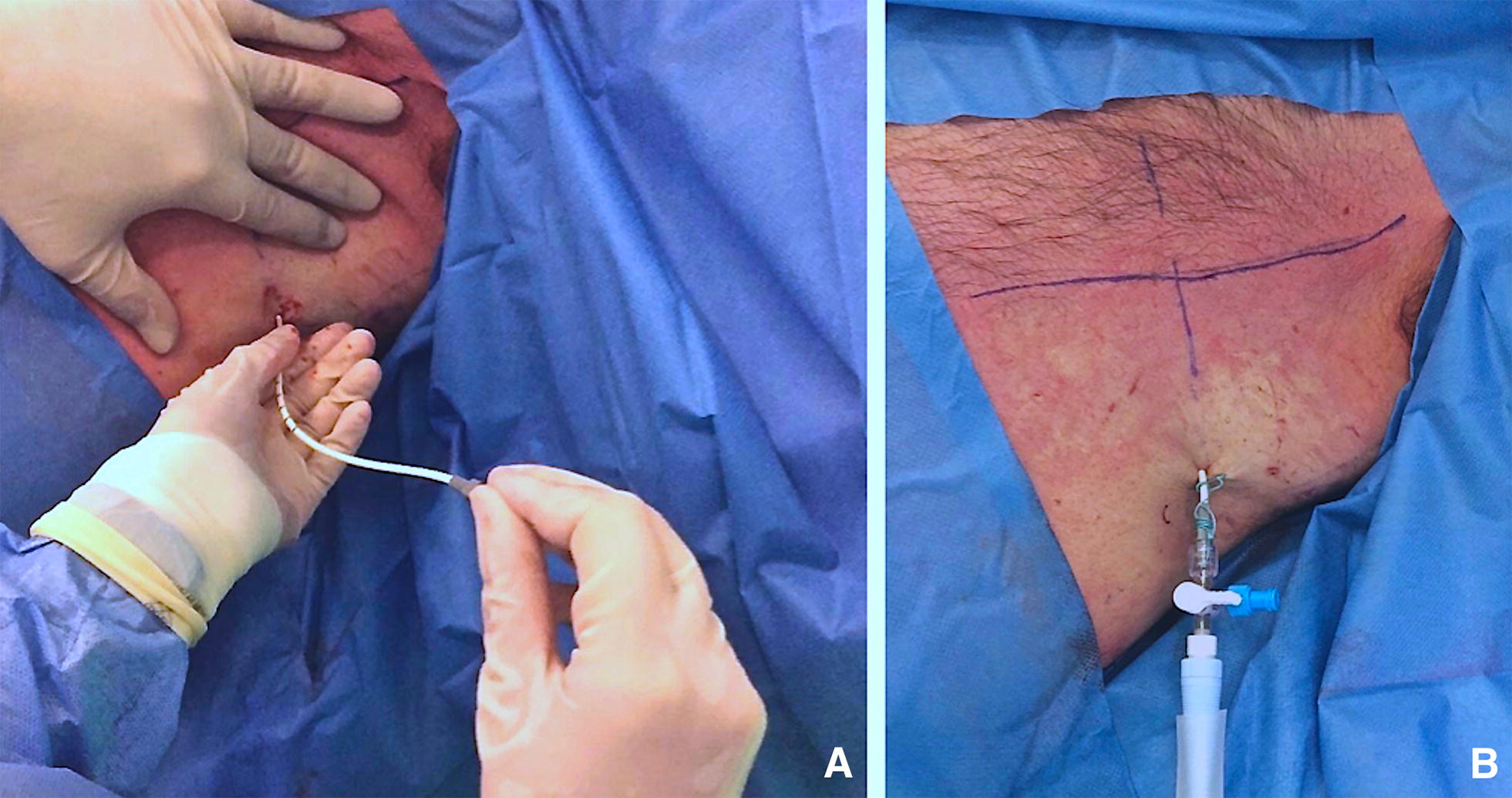
**A** The pigtail is passed over the guidewire, making sure that the last side hole is within the pleural space. Remove the guidewire, leaving the pigtail catheter in place. After the guidewire has been removed, the drain is connected to the drainage system. **B** Suture the pigtail to the chest wall in a manner similar to conventional chest tubes
F. Ultrasound confirmation. At the end of the procedure, it is mandatory to perform a complete bilateral lung ultrasound scan to exclude possible complications (e.g., pneumothorax) and confirm the pigtail position.

A step-by-step guide for pigtail insertion effusion is provided in Additional file 4: Video S4. It includes ultrasound guidance, insertion of the introducer needle to ultrasound confirmation of the pigtail catheter within PLEFF.

### Complications and side-effects

The pigtail catheter is a smaller drain (8–14F) compared to traditional chest tubes. Pigtail catheters insertion includes the following complications:

- Pneumothorax;
- Dry tap;
- Subdiaphragmatic insertion (injury to the diaphragm, liver or spleen with significant haemorrhage);
- Laceration of adjacent structures (pleural or cardiac laceration);
- Intercostal artery laceration;
- Catheter malposition;
- Catheter dislodgement or kinking;
- Catheter blockage;
- Wound infection and empyema;
- Re-expansion pulmonary edema.

Insertional complications, dry taps and procedure failure are less common with ultrasound guidance [11].

The main complications linked to small-bore catheters are blockade, dislodgement, malposition, and kinking [30]. The disconnection of the tubing system represents another possible complication. For infected pleural-fluid effusions, the efficacy and safety of intrapleural fibrinolytic agents are still debatable [11, 31].

Wound infection and empyema may occur if the chest tube (a foreign object) introduces bacteria into the pleural space.

The etiopathogenesis of the re-expansion oedema (RPO) is relatively unknown. Over the years different hypotheses have arisen about increased permeability of pulmonary blood vessels damaged by fast re-expansion of lung tissue [32], blood vessels damaging by free oxygen radicals generated by reperfusion of ischemic lung [33], pulmonary hydrostatic pressure caused by increased venous return, pressure-induced disruption of the alveolar capillaries and altered lymphatic clearance [34]. Mokotedi postulated that RPO could be due to decreased pleural and intrathoracic pressure and left ventricular afterload increased after pleural drainage with a detrimental effect on LV performance in patient with more than moderate compromised LV systolic function [35]. Rapid lung re-expansion in the following settings [36] has been described as a risk factor:

- Young patients 20–39 aa [37]
- Large pneumothorax
- Large volume pleural drainage (> 3 L) or high negative pressure suction (more than − 20 cm H_2_O) [38]
- Lung collapsed for over 7 days.

Due to the fact that the etiology of re-expansion pulmonary oedema is unclear, there is concern that re-expansion pulmonary oedema may occur if larger volumes of fluid are withdrawn. Despite BTS guidelines, consensus statement of American College of Chest Physicians and most authors advise to drain no more than 1–1.5 L of fluid at one time, the amount of fluid safely removed continues to be debated as in several studies volumes greater than 1.5 L up to 6 L were safely aspirated.

Cause the RPO mortality rate has been quoted as high as 20%, preventive strategies include limiting drainage of pleural fluid, if the patient reports chest pressure or discomfort during thoracentesis, and using low negative pressure (less than − 20 cm H_2_O) for suction with serial measures of pleural pressure can lead to prevention and early recognition of complication. Pleural manometry is not currently in clinical practice and there are no randomised controlled trials. Two important warning signs to end the aspiration are if the patient develops a cough or complains of chest discomfort [11].

Remember that pigtails can easily be drawn back, but cannot be inserted farther in after the procedure is completed.

## Conclusions

Placement of a pigtail catheter is a therapeutic manoeuvre in the presence of a pleural effusion that may noticeably improve gas exchange and respiratory mechanisms (i.e., respiratory system compliance, increase of functional residual capacity). Ultrasound guidance allows the operator to increase the rate of success of the procedure and reduce its associated risks. Consequently, usage of the ultrasound guidance during pleural drainage has become mandatory. Training programs, using appropriate manikins [39], must be encouraged to teach residents and clinicians how to use US during a pleural procedure.

## Additional files

**Additional file 1: Video S1.** Ultrasound allows for the visualisation of the vascular bundles, thus minimising the risk of damage to nerves and vessels. As shown, the intercostal neurovascular bundle is easily visible below the lower margin of the rib.

**Additional file 2: Video S2.** Ultrasound guidance allows the operator to decide where to insert the pigtail. The best puncture site is the place where the operator best visualises each anatomical structures (i.e., diaphragm, pleural, organs) and where the operator can measure the maximum distance between visceral and parietal pleural (increasing the safety margin). The probe should be used in the transverse position between two ribs. The probe marker is facing the patient’s head (on the right of the screen).

**Additional file 3: Video S3.** Check the position of the guidewire using thoracic ultrasound (TUS) before introducing the dilator. As shown, the guidewire is positioned correctly within the pleural effusion. The operator can insert the dilator.

**Additional file 4: Video S4.** The use of ultrasound allows identification of the best puncture site and for recheck, at all times, the correct position of the devices used. In this video, we can see all the procedure previously descripted.

## Abbreviations

TUS: thoracic ultrasound
PLEFF: pleural effusion
ICU: intensive care unit
CT: computed tomography
US: ultrasound
PEEP: positive end-expiratory pressure
PRF: pulse repetition frequency
MHz: megahertz
RPE: re-expansion pulmonary edema
